# Infliximab associated with faster symptom resolution compared with corticosteroids alone for the management of immune-related enterocolitis

**DOI:** 10.1186/s40425-018-0412-0

**Published:** 2018-10-11

**Authors:** Daniel H Johnson, Chrystia M Zobniw, Van A Trinh, Junsheng Ma, Roland L Bassett, Noha Abdel-Wahab, Jaime Anderson, Jennifer E Davis, Jocelyn Joseph, Marc Uemura, Ali Noman, Hamzah Abu-Sbeih, Cassian Yee, Rodabe Amaria, Sapna Patel, Hussein Tawbi, Isabella C Glitza, Michael A Davies, Michael K Wong, Scott Woodman, Wen-Jen Hwu, Patrick Hwu, Yinghong Wang, Adi Diab

**Affiliations:** 1 Section of Rheumatology and Clinical Immunology, Department of General Internal Medicine, The University of Texas MD Anderson Cancer Center, Houston, TX USA; 2 Department of Rheumatology and Rehabilitation, Faculty of Medicine, Assiut University Hospitals, Assiut, Egypt; 3 Department of Biostatistics, The University of Texas MD Anderson Cancer Center, Houston, TX USA; 4 Department of Epidemiology, Division of Cancer Prevention and Population Sciences, The University of Texas MD Anderson Cancer Center, Houston, TX USA; 5 Department of Gastroenterology, Hepatology, and Nutrition, Division of Internal Medicine, The University of Texas MD Anderson Cancer Center, Houston, TX USA

**Keywords:** Infliximab, Immune-related enterocolitis, Immune checkpoint inhibitors

## Abstract

**Background:**

Immune-related enterocolitis (irEC) is the most common serious complication from checkpoint inhibitors (CPIs). The current front-line treatment for irEC, high-dose corticosteroids (CS), have significant side effects and prolonged therapy may reduce CPI-anti-tumor activity. Early addition of TNF-α inhibitors such as infliximab (IFX) may expedite symptom resolution and shorten CS duration. Thus, we conducted the first retrospective study, to our knowledge, evaluating symptom resolution in patients with irEC treated with and without IFX.

**Methods:**

Data were collected from the medical records of patients diagnosed with irEC. The primary endpoint was time to symptom resolution for irEC for cases managed with IFX plus CS (IFX group) versus CS alone (CS group). Duration of CS, overall survival (OS), and time to treatment failure (TTF) were secondary endpoints.

**Results:**

Among 75 patients with irEC, 52% received CS alone, and 48% received IFX. Despite higher grade colitis in the IFX group (grade 3/4: 86% vs. 34%; *p* < 0.001), median times to diarrhea resolution (3 vs. 9 days; *p* < 0.001) and to steroid titration (4 vs. 13 days; *p* < 0.001) were shorter in the IFX group than in the CS group without a negative impact on TTF or OS. Total steroid duration (median 35 vs. 51 days; *p* = 0.150) was numerically lower in the IFX group.

**Conclusions:**

Despite higher incidence of grade 3/4 colitis, IFX added to CS for the treatment of patients with irEC was associated with a significantly shorter time to symptom resolution. The data suggest that early introduction of IFX should be considered for patients with irEC until definitive prospective clinical trials are conducted.

**Electronic supplementary material:**

The online version of this article (10.1186/s40425-018-0412-0) contains supplementary material, which is available to authorized users.

## Background

Checkpoint inhibitors (CPIs) have improved overall survival (OS) for patients with various malignancies [1–5]. Currently available CPIs target the cytotoxic T-lymphocyte antigen 4 (anti–CTLA-4 agents include ipilimumab and tremelimumab), programmed death 1 (anti–PD-1 agents include pembrolizumab and nivolumab), and programmed death ligand 1 (anti–PD-L1 agents include atezolizumab, avelumab, and durvalumab). Newer agents continue to emerge, expanding the therapeutic applications of CPIs in cancer management. However, CPIs can cause severe immune-related adverse events, among which immune-related enterocolitis (irEC) is the most common serious complication [6].

IrEC extensively affects the gastrointestinal tract. If irEC is not treated promptly, it can lead to bowel perforation, sepsis, and death [7]. Although toxicity management algorithms have been shown to reduce the incidence of serious gastrointestinal complications from irEC [8], the rate of mortality due to irEC remains near 1% [7, 9]. Despite the expanding role of CPIs in cancer therapy and the frequency of CPI-related adverse events, there remains a paucity of data regarding the optimal management of these immune related adverse events.

The recommended front-line treatment for irEC is high-dose corticosteroids (CS) (methylprednisolone at 1–2 mg/kg or its equivalent) [6, 7, 10–12]. Protracted high-dose CS may precipitate serious complications, e.g., hyperglycemia, hepatic steatosis, muscle atrophy, opportunistic infection, and death [13]. Tumor necrosis factor-alpha (TNFα), a critical co-factor for mucosal Th1 cytokine production, plays a central role in the pathogenesis of mucosal inflammation in inflammatory bowel disease [14, 15]. TNFα-blocking agents, including infliximab (IFX), have revolutionized the treatment of moderate to severe inflammatory bowel disease and have been reported to induce rapid symptom resolution and to facilitate steroid tapering even in CS-refractory irEC cases [16]. The early addition of IFX in irEC management may promptly reverse gastrointestinal symptoms, avoid perforation or death, and spare patients the toxicities of prolonged steroid exposure. Thus, we conducted a retrospective study to compare the efficacy of IFX plus CS with that of CS alone for the management of irEC.

## Methods

### Study design

Adult cancer patients treated with CPIs (ipilimumab, tremelimumab, nivolumab, pembrolizumab, atezolizumab, or ipilimumab-nivolumab in combination) between January 1, 2012, and June 30, 2017, were identified by review of the institutional pharmacy database at MD Anderson Cancer Center. Included in our analysis were patients for whom there was adequate documentation of the timing of our primary endpoints in the medical records. Patients who received IFX and CS (dexamethasone, methylprednisolone, and prednisone) as well as those who received CS alone were identified. Their records were then reviewed to identify the indication for those treatments, and patients who received IFX and/or CS for a reason other than irEC were excluded from further analysis. All treatments for irEC were per the treating oncologist’s discretion. Baseline characteristics at the time of irEC diagnosis, including age, sex, co-morbidities, cancer type, and CPI regimen, were collected. Stool studies for infectious cause, endoscopic and radiographic evaluations, immunosuppressant regimens, CS duration, number of IFX doses, and treatment side effects were documented.

In this study, irEC was defined as diarrhea requiring immunosuppressants in the setting of CPI therapy and was graded according to the Common Terminology Criteria for Adverse Events (CTCAE) version 4.0 based solely on diarrhea. CTCAE classify and grade diarrhea and colitis as two separate entities. In the setting of CPI therapy, diarrhea is a widely accepted surrogate for irEC and is treated as presumed irEC [8]. We graded irEC solely on the basis of diarrhea because other irEC-associated signs and symptoms, such as abdominal pain and presence of mucus or blood in stool, were not as consistently documented in patients’ charts as was frequency of loose stools.

The primary endpoint, time to symptom resolution, was defined in two ways: the time from initiation of IFX and/or CS to either (i) complete resolution of diarrhea (absence of diarrhea) or (ii) initiation of steroid titration due to improvement of irEC symptoms as indicated by documentation in the electronic medical record. Although the timing of initiation of steroid titration depends on the physician’s discretion, we considered this co–primary endpoint another surrogate for resolution of colitis symptoms. Among the secondary endpoints was CS treatment duration, defined as the time from initiation of IFX and/or CS to the last dose of CS.

To assess the relative impact of these immunosuppressive therapies on CPI clinical activity, we evaluated OS and time to treatment failure (TTF), defined as the time from the initiation of CPI to oncologic therapy change due to disease progression among patients with stage IV melanoma only.

### Statistical analysis

Patient characteristics were reported as percentages for categorical variables and as medians for continuous variables. Categorical and continuous variables were compared between groups by Fisher exact test and Kruskal-Wallis test, respectively. The log-rank and Renyi tests were used to compare survival curves between treatment groups. Forest plots based on results from gamma generalized linear models (with log link function) were used to display treatment differences regarding the time to diarrhea resolution and time to steroid titration within subgroups of given variables. For each subgroup, the treatment multiplicative effect and its 95% confidence intervals (CI) were estimated. Safety assessments were descriptive in nature. Statistical analyses were performed using R version 3.3.1. All statistical tests used a significance level of 5%. No adjustments were made for multiple testing.

## Results

### Patient characteristics

**Fig. 1:**
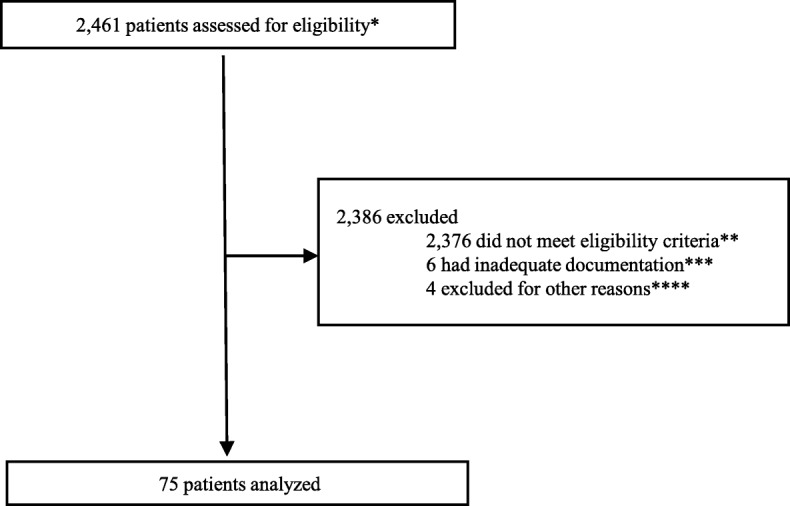
Patient Selection. *Patients with malignancy who receivied CPIs. **Eligibility criteria: (1) received IFX and/or CS (dexamethasone, methylprednisolone, and prednisone) and (2) indication for IFX and/or CS definitively for irEC. ***Inadequate documentation of timing of primary endpoints (symptoms resolution). ****Did not meet primary endpoint of symptom resolution or died before resolution of colitis (3 in CS group, 1 in IFX group)

A total of 75 patients with irEC were included in the study (Fig. 1). Of these, 36 were treated with IFX plus CS (IFX group), and 39 received CS only (CS group). The baseline patient characteristics are shown in Table 1. The median age at the development of irEC was 63 years in patients treated with CS alone and 61 years in patients treated with IFX plus CS. Most patients were male (67%) and had a diagnosis of melanoma (71%). The baseline characteristics were generally balanced between the IFX and the CS groups; however, there were significantly more men in the IFX group than in the CS group.

**Table 1:** Baseline Characteristics

Characteristic	Overall (*N* = 75)	Infliximab + CS (*N* = 36)	CS Alone (*N* = 39)	*P* Value
Median Age (Interquartile Range)		63 years (50–71 years)	61 years (52–72 years)	0.633
Sex, No. (%)				
Male	50 (66.7)	29 (80.6)	21 (53.8)	0.016
Female	25 (33.3)	7 (19.4)	18 (46.2)	0.016
Cancer Type, No. (%)				
Melanoma	53 (70.7)	29 (80.6)	24 (61.5)	0.167
Genitourinary^a^	15 (20.0)	7 (19.4)	8 (20.5)	
Head and Neck Cancer	1 (1.3)	0	1 (2.6)	
Leukemia	1 (1.3)	0	1 (2.6)	
Lung Cancer	2 (2.7)	0	2 (5.1)	
Other^b^	3 (4.0)	0	3 (7.7)	
Immunotherapy Type, No. (%)				
Ipilimumab	45 (60.0)	21 (58.3)	24 (61.5)	0.834
Ipilimumab + Nivolumab	12 (16.0)	6 (16.7)	6 (15.4)	
Nivolumab	4 (5.3)	1 (2.8)	3 (7.7)	
Pembrolizumab	13 (17.3)	7 (19.4)	6 (15.4)	
Tremelimumab	1 (1.3)	1 (2.8)	0	
Symptoms at Onset, No. (%)				
Diarrhea	73 (97.3)	36 (100)	37 (94.9)	0.494
Nausea	21 (28.0)	8 (22.2)	13 (33.3)	0.315
Vomiting	11 (14.7)	3 (8.3)	8 (20.5)	0.195
Abdominal Cramps	32 (42.7)	13 (36.1)	19 (48.7)	0.351
Blood in Stool	22 (29.3)	14 (38.9)	8 (20.5)	0.127
Mucus in Stool	2 (2.7)	0	2 (5.1)	0.494
Elevated WBC Counts	3 (4.0)	1 (2.8)	2 (5.1)	1
Pathology, No. (%)				
Positive	37 (49.3)	20 (55.6)	17 (43.6)	0.534
Negative	6 (8.0)	2 (5.6)	4 (10.3)	
Not Tested	32 (42.7)	14 (38.9)	18 (46.2)	

^a^Includes renal cell carcinoma, urothelial carcinoma, and prostate cancer

^b^Includes anal squamous cell cancer, pancreatic cancer, and glioblastoma

*CS* corticosteroids, *WBC* White blood cells

The clinical characteristics of the irEC cases are presented in Table 1. CPIs utilized were similar between the two groups; ipilimumab was the most commonly used agent. Other than diarrhea, the most frequent presenting symptoms were abdominal cramps (43%), bloody stools (29%), and nausea (28%). Stool studies were performed in 60 patients. *Clostridium difficile* infection was found in three patients in each group. Endoscopic evaluation was performed in 42 (56%) patients, and biopsy-proven colitis was found in 37 (49%). In the 5 patients who underwent endoscopy without a tissue diagnosis of colitis, there was either clinically evident colitis on endoscopy or reports were unavailable. Pathology findings consistent with colitis were seen in 20 (56%) patients in the IFX group and 17 (44%) patients in the CS group. Computerized tomography was performed in 42 (56%) patients, and wall thickening was present in 21 (28%). In the IFX group, one to three doses of IFX were administered; 26 (72%) patients had one dose, eight (22%) patients had two doses, and two (6%) patients had three doses. IFX was administered within a median of 8 days after CS initiation. Every patient had CPI therapy discontinued at the time of diagnosis of irEC. There were 18 patients re-challenged within 6 months of completing steroids and/or infliximab, 12 patients (33%) in the IFX group and 6 patients (15%) in the CS group.

At the onset of irEC, grade 3/4 colitis was documented with numerically higher frequency in the IFX group (61%) than in the CS group (34%) (*p* = 0.077). Initial CS therapy alone did not seem to impact the severity of irEC in the IFX group. In fact, the grade of colitis significantly worsened from the onset of diarrhea to the time of IFX administration; grade 3/4 colitis was seen in 86% of the IFX group (by the time of IFX initiation) but only 34% of the CS group (*p* < 0.001) (Table 2).

**Table 2:** Grade of Colitis

	CS Alone (*N* = 38)]	Infliximab + CS (N = 36)	
Measure	At CS Initiation	At CS Initiation	At IFX Initiation
Colitis Grade*, No. (%)			
1	8 (21.1)	4 (11.1)	0
2	17 (44.7)	10 (27.8)	5 (13.9)
3/4	13 (34.2)	22 (61.1)	31 (86.1)
*P* Value		0.077	< 0.001**

*Per Common Terminology Criteria for Adverse Events 4.0 and based solely on diarrhea

**Compared with CS group at CS initiation

*CS* corticosteroids

### Efficacy

**Fig. 2:**
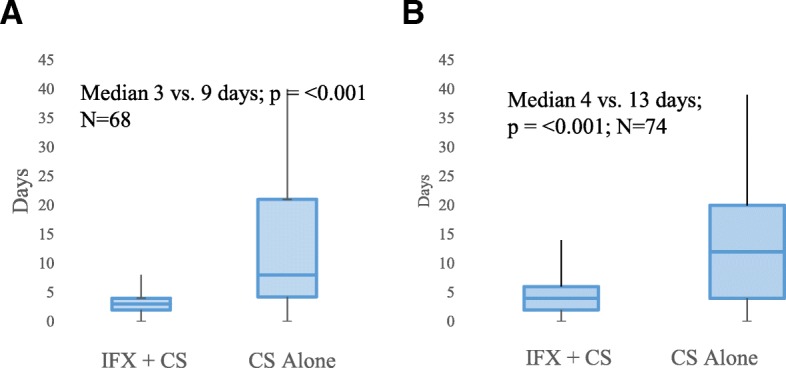
Box plots of association between treatment for irEC and time to irEC symptom resolution Symptom resolution defined as (**a**) time to diarrhea resolution (Median 3 days vs. 9 days [IQR 5·75–22·0]; *p* = < 0.001; *N* = 68) and (**b**) time to initiation of steroid titration (Median 4 days [IQR 2·0–6·0] vs. 13 days [IQR 5·5–20·5]; (*p* = < 0.001; *N* = 74)

**Fig. 3:**
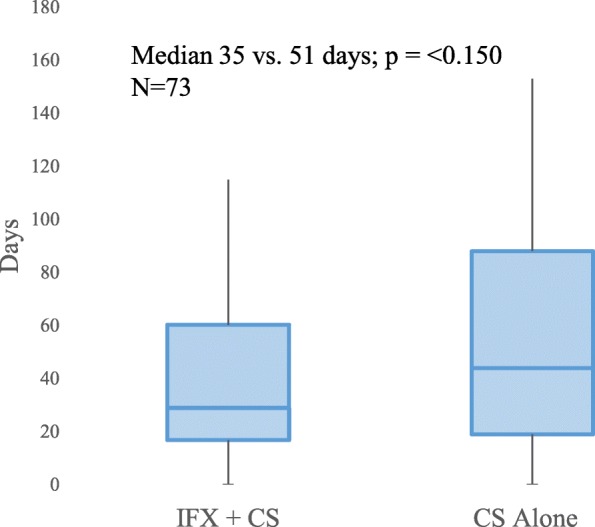
Box plot of association between treatment for irEC and total duration of corticosteroids. Median 35 days [IQR 24·3–64·8] vs. 51 days [IQR 27·0–94·0]; *p* = < 0.150; *N* = 73

Among the 75 patients included in the study, 68 patients had documentation of the timing of diarrhea resolution; 73 patients had documentation of the timing of steroid titration initiation. All of these 73 patients had documentation in the EMR that the reason for initiation of steroid titration was improvement in irEC symptoms. The IFX group had significantly shorter times to diarrhea resolution (median 3 days vs. 9 days; *p* < 0.001) and to steroid titration (median 4 days vs. 13 days; *p* < 0.001) compared with the CS group (Fig. 2). The IFX group also had numerically shorter CS duration (median 35 days vs. 51 days; *p* = 0.150) than did the CS group (Fig. 3).

**Fig. 4:**
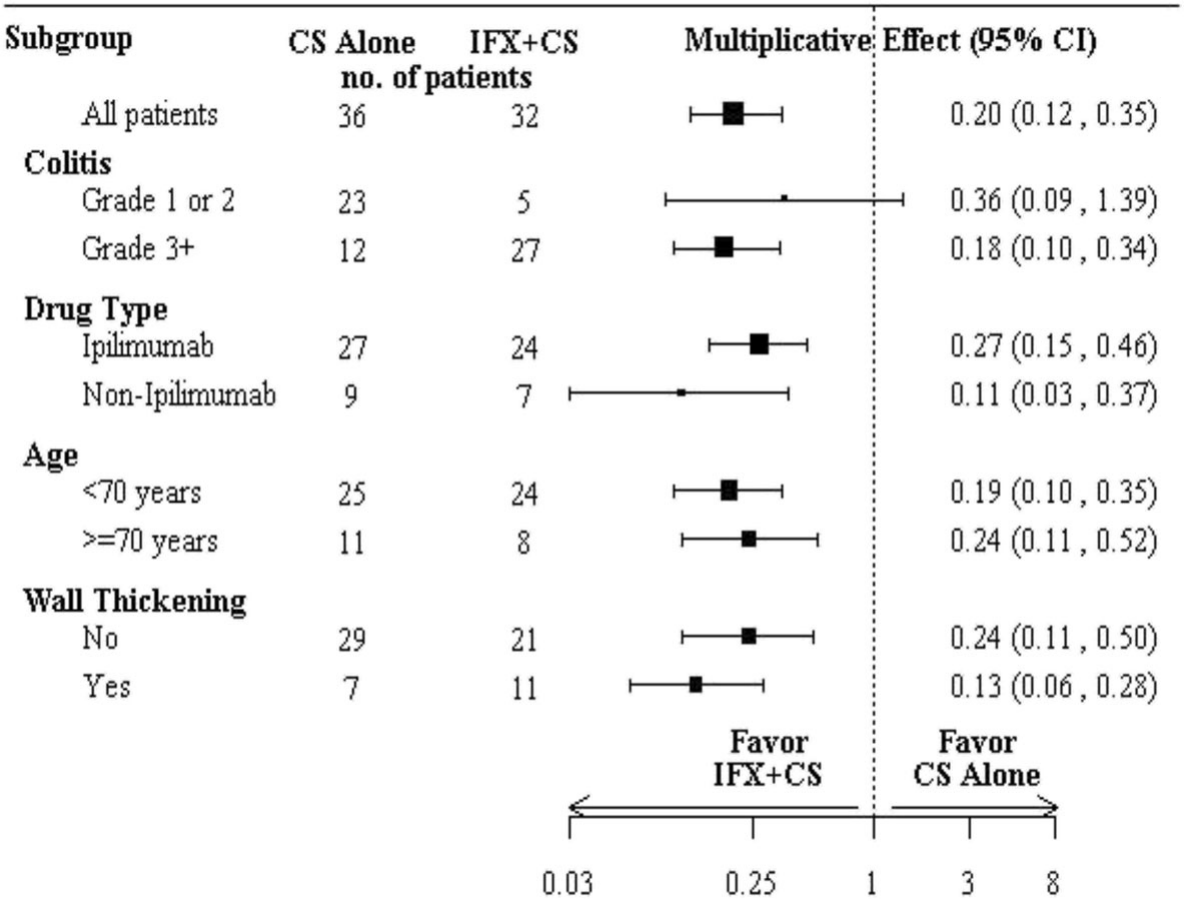
Univariate subgroup analysis for the multiplicative effect of irEC treatment (IFX + CS vs. CS alone) on time to diarrhea resolution. *CS* Corticosteroids, *IFX* Infliximab

Shorter time to diarrhea resolution in the IFX group than the CS group was also observed in all clinically relevant subgroups, where patients were stratified by colitis grade, patient age, CPI regimen, and presence of colonic wall thickening on radiographic imaging (Fig. 4). Bowel perforation occurred in one patient in the IFX group; the perforation occurred within 24 h of IFX initiation and hence was more likely due to inadequate or unsuccessful rescue therapy than to IFX therapy.

**Fig. 5:**
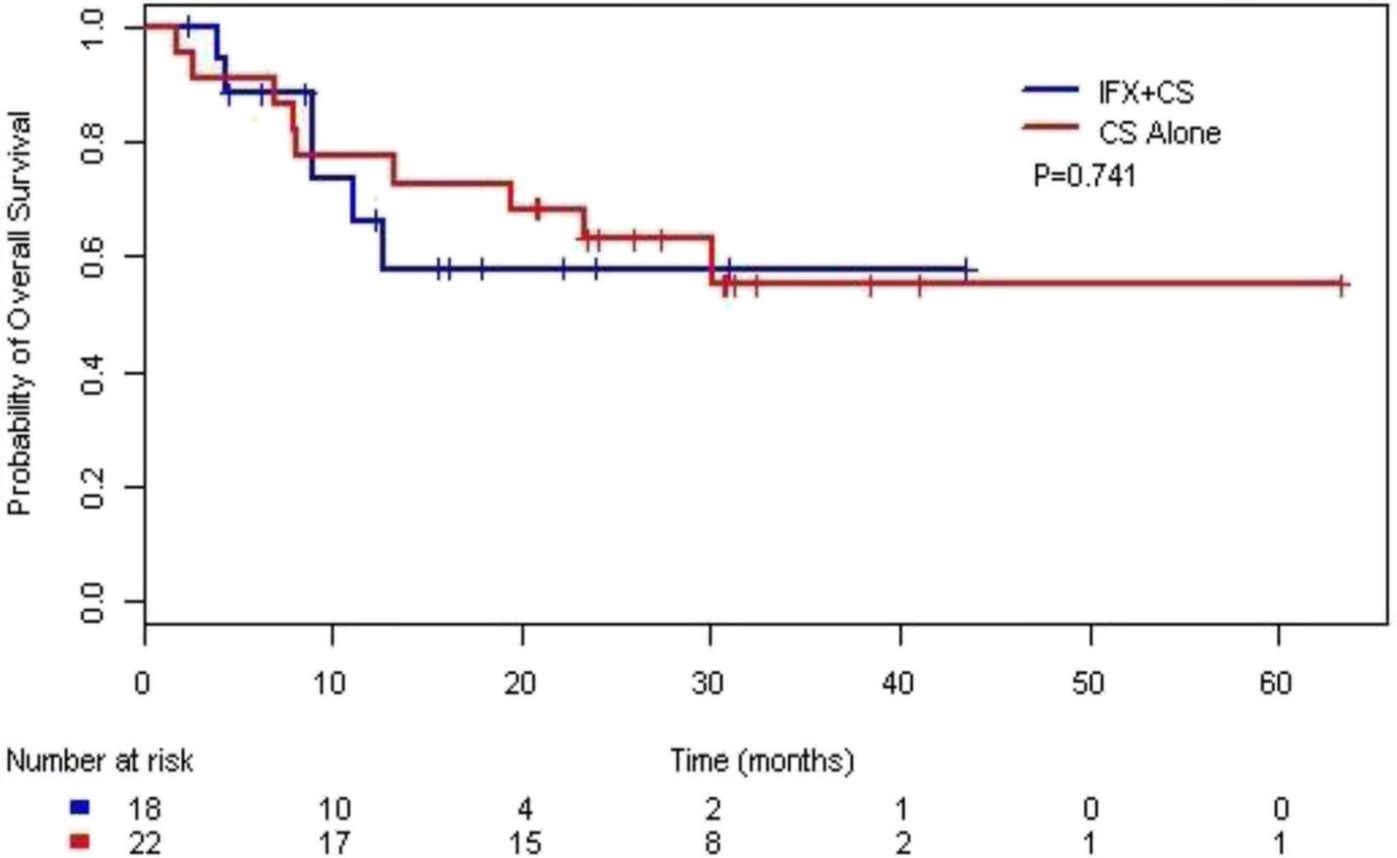
Kaplan-Meier analysis of overall survival in patients with stage IV melanoma. Median not reached in IFX or CS group. Median follow-up 26 months

TTF and OS analyses were carried out for 40 patients with stage IV melanoma, of whom 18 and 22 patients were treated in the IFX and CS groups, respectively. At a median follow-up duration of 26.0 months, the median TTF was 9.0 months (95% CI 5.6 months–not reached) in the IFX group and 12.5 months (95% CI 5.8 months–not reached) in the CS group (Additional file 1). Median OS was not reached in either group (Fig. 5). The differences in TTF and OS between the two treatment groups were not statistically significant.

### Safety

There were no statistically significant differences in the rates of CS-related side-effects between the IFX group (61%) and the CS group (46%; *p* = 0.249) (Additional file 1). The most common adverse events associated with CS use included hyperglycemia, muscle weakness, and edema. Additional infectious complications occurred in two patients in the IFX group and included herpes virus infection and cytomegalovirus viremia.

## Discussion

To our knowledge, this study is the first retrospective case series to compare the efficacy of IFX plus CS and that of CS alone in patients with irEC. Our study demonstrates that patients treated in the IFX group had a statistically significantly shorter time to symptom resolution than did those in the CS group. In the IFX group, median times to diarrhea resolution and to steroid titration were 3 days and 4 days, respectively, in contrast to 9 days and 13 days, respectively, in patients who received CS alone. Intriguingly, we report a faster time to symptom resolution with the addition of IFX despite more severe irEC in the IFX group. Faster symptom resolution reflects rapid reversal of colonic tissue inflammation that could otherwise deteriorate into perforation and even death without effective management. Prompt symptom resolution may profoundly improve patients’ quality of life, shorten time of hospitalization, and decrease healthcare costs. Furthermore, there were a higher proportion of patients with grade 3/4 colitis at the time of IFX administration than at the time of symptom onset. This signifies multiple patients in the IFX group with grade 1 or 2 irEC at diagnosis had deteriorated to grade 3/4 while on CS alone, suggesting that the CS given prior to IFX initiation was not having a beneficial impact on symptom resolution. For this reason, we used initiation of IFX as the starting time-point for the primary endpoints. A history of grade 3/4 immune-related adverse events is a common exclusion criterion for many immunotherapy trials, directly limiting a patient’s treatment options. We believe that aggressive, upfront treatment of irEC before symptoms progress is clinically relevant and important for patient outcomes.

Diarrhea and colitis are more frequent and severe with anti-CTLA4 agents (all grades: 30%; grade 3–4: 10–16%) than with agents targeting the PD-1/PD-L1 axis (all grades: 11–16%; grade 3–4: 1–2%) [6, 7, 10, 11]. In particular, these agents in combination have higher rates of immune related adverse events with diarrhea being most common (all grades: 45%; grade 3–4 11%) [17]. The majority of patients reported here (57–86%) received ipilimumab as monotherapy or in combination with a PD-1 inhibitor. Although the use of ipilimumab monotherapy front-line for metastatic melanoma (MM) or in the adjuvant setting for resected Stage III melanoma has been largely replaced by PD-1 inhibitors, we feel that CTLA-4 inhibitors, particularly in combination with PD-1 blockade, will continue to be widely used in multiple cancer types. Ipilimumab in combination with nivolumab is approved for front-line MM, and after progression on PD-1 inhibitors, ipilimumab continues to be the standard second line immunotherapy. In Checkmate-214, a phase III, randomized study evaluating the combination of nivolumab and ipilimumab vs. sunitinib in patients with treatment-naïve, intermediate or poor risk metastatic renal cell carcinoma, there was a significant improvement in OS and response rate [18]. Results from Checkmate-227, a phase III study evaluating the same regimen as first line for advanced non-small cell lung cancer patients with a high tumor mutational burden, demonstrated a significantly improved progression free survival compared to standard of care chemotherapy [19]. With approval of ipilimumab with nivolumab for front-line treatment in melanoma and renal cell carcinoma and likely approval for high mutational load non-small cell lung cancer, we believe that optimization of management for anti-CTLA-4 induced colitis will continue to be relevant.

In this study, IFX use was associated with a numerically shorter median duration of steroid exposure (35 days vs. 51 days; *p* = 0.150) without a negative impact on TTF or OS in the patients with metastatic melanoma. Most patients only required one dose of IFX (72%) to achieve symptom resolution. The recommended front-line treatment for grade 3/4 irEC is high-dose CS [6, 10]. Many patients require prolonged CS exposure, and some cases can even be refractory to CS. Thus, inflammatory cytokine blockade such as IFX may be not only an important treatment for steroid-refractory irEC but also potentially an effective steroid-sparing strategy to reduce serious CS-related complications. We previously reported a patient who developed atrial fibrillation secondary to prolonged CS use for severe ipilimumab-related rheumatoid arthritis. The CS were discontinued, and the patient was started on tocilizumab (an anti–interleukin-6R monoclonal antibody). The arthritis resolved, and the patient’s metastatic melanoma was in durable remission at last follow-up [20].

It has been suggested that immunosuppressive therapy for immune-related adverse events in patients with advanced melanoma receiving CPIs do not affect TTF or OS [21]. However, these findings may have been confounded by higher response rates in patients experiencing immune-related adverse events [22, 23]. We believe that significantly prolonged steroid exposure, especially when coupled with CPI suspension, may negatively impact the clinical benefit of immunotherapy. We realize that without a prospective, pre-planned CS titration protocol, it is difficult to draw definitive conclusions from these data about the efficacy of IFX as a steroid-sparing treatment. However, the numerically shorter CS duration that we observed in the IFX group is an encouraging finding with potential clinical implications.

Our results demonstrate that adding IFX, at limited doses, to CS for irEC treatment had no significant effect on OS or TTF in patients with stage IV melanoma. Our cohort of patients with irEC received immunotherapies for a variety of cancer types at different stages (Table 1), which would confound the analysis of the effect of systemic immunosuppression on CPI therapeutic benefit. For this reason we chose to include only patients with stage IV melanoma in our OS and TTF analysis. The resulting limited sample size along with high censoring due to short follow-up (median follow-up of 26 months) were major limitations to this analysis. TTF has been used as a surrogate endpoint for progression-free survival [21]; however, this endpoint could be confounded by multiple factors in a retrospective analysis. For example, the initiation of the next line of cancer-directed therapy, in particular immunotherapy, may have been delayed by unresolved irEC and ongoing CS treatment. To that point, the faster time to irEC symptom resolution and shorter CS duration we saw in association with IFX could result in earlier initiation of the next line of therapy.

TNFα is an inflammatory cytokine that plays contrasting roles in immunology. In rheumatoid arthritis or inflammatory bowel disease, TNFα blockade can successfully lead to clinically beneficial immune suppression. However, in other autoimmune disorders, such as multiple sclerosis and lupus nephritis, TNFα deficiency or blockade can exacerbate inflammation and precipitate disease flare [24, 25]. Similarly, in tumor immunology, TNFα can mediate opposing effects. While TNFα has traditionally been known as a stimulatory cytokine that is important for the cytotoxic effector T cell response, a growing body of evidence has described its role in promoting immune suppression by facilitating the proliferation and function of regulatory immune cells (T regulatory cells and myeloid-derived suppressor cells) [26, 27]. These immunosuppressive features of TNFα were associated with chronic and prolonged exposure to the cytokine [27] In a recent preclinical study, TNFα blockade was shown to significantly enhance tumor immunity and overcome resistance to anti–PD-1 and adoptive T cell therapy [28]. Our data demonstrate that short-term IFX use (1–3 doses) did not compromise OS and clinical benefit from CPIs. Interestingly, there is a phase Ib clinical trial evaluating the safety and efficacy of anti-TNFα given concomitantly with ipilimumab and nivolumab in patients with advanced melanoma (NCT03293784).

## Conclusion

Although this is a retrospective, single-institution experience, our study provides much-needed data supporting the early use of IFX to hasten irEC symptom resolution, particularly in patients with high-grade colitis. The recent termination of the only prospective trial evaluating IFX plus CS versus CS alone for irEC (NCT02763761) due to insufficient enrollment further highlights the relevance of these data. It is important to identify more targeted approaches to treat irEC that mechanistically do not inhibit the anti-tumor immune response. However, until newer approaches for irEC are available, we recommend the early addition of IFX for irEC.

## Additional file

Additional file 1: **Figure S1.** Kaplan-Meier analysis of time to treatment failure in patients with stage IV melanoma. Median TTF was 9.0 months (95% CI 5.6 months–not reached) in the IFX group and 12.5 months (95% CI 5.8 months–not reached) in the CS group. Median follow-up 26 months. (DOCX 48 kb)
