# Correction to: Infliximab associated with faster symptom resolution compared with corticosteroids alone for the management of immune-related enterocolitis

**DOI:** 10.1186/s40425-018-0469-9

**Published:** 2019-04-17

**Authors:** Daniel H. Johnson, Chrystia M. Zobniw, Van A. Trinh, Junsheng Ma, Roland L. Bassett Jr, Noha Abdel-Wahab, Jaime Anderson, Jennifer E. Davis, Jocelyn Joseph, Marc Uemura, Ali Noman, Hamzah Abu-Sbeih, Cassian Yee, Rodabe Amaria, Sapna Patel, Hussein Tawbi, Isabella C. Glitza, Michael A. Davies, Michael K. Wong, Scott Woodman, Wen-Jen Hwu, Patrick Hwu, Yinghong Wang, Adi Diab

**Affiliations:** 1 Department of Melanoma Medical Oncology, The University of Texas MD Anderson Cancer Center, Houston, TX USA; 2 Division of Pharmacy, Clinical Section, The University of Texas MD Anderson Cancer Center, Houston, TX USA; 3 Department of Biostatistics, The University of Texas MD Anderson Cancer Center, Houston, TX USA; 4 Section of Rheumatology and Clinical Immunology, Department of General Internal Medicine, The University of Texas MD Anderson Cancer Center, Houston, TX USA; 5 Department of Rheumatology and Rehabilitation, Faculty of Medicine, Assiut University Hospitals, Assiut, Egypt; 6 Department of Epidemiology, Division of Cancer Prevention and Population Sciences, The University of Texas MD Anderson Cancer Center, Houston, TX USA; 7 Department of Gastroenterology, Hepatology, and Nutrition, Division of Internal Medicine, The University of Texas MD Anderson Cancer Center, Houston, TX USA

**Correction to: J Immunother Cancer**

10.1186/s40425-018-0412-0

Following publication of the original article [1], the authors reported an error in their listed affiliations. This error was introduced during typesetting and the publisher apologizes to readers and the authors for the inconvenience.

In this Correction the authors are listed with their correct affiliations below.

Daniel H Johnson^1^, Chrystia M Zobniw^2^, Van A Trinh^2^, Junsheng Ma^3^, Roland L Bassett Jr^3^, Noha Abdel-Wahab^4,5^, Jaime Anderson^2^, Jennifer E Davis^6^, Jocelyn Joseph^2^, Marc Uemura^1^, Ali Noman^7^, Hamzah Abu-Sbeih^7^, Cassian Yee^1^, Rodabe Amaria^1^, Sapna Patel^1^, Hussein Tawbi^1^, Isabella C Glitza^1^, Michael A Davies^1^, Michael K Wong^1^, Scott Woodman^1^, Wen-Jen Hwu^1^, Patrick Hwu^1^, Yinghong Wang^7^ and Adi Diab^1^

^1^Department of Melanoma Medical Oncology, The University of Texas MD Anderson Cancer Center, Houston, TX, USA

^2^Division of Pharmacy, Clinical Section, The University of Texas MD Anderson Cancer Center, Houston, TX, USA

^3^Department of Biostatistics, The University of Texas MD Anderson Cancer Center, Houston, TX, USA

^4^Section of Rheumatology and Clinical Immunology, Department of General Internal Medicine, The University of Texas MD Anderson Cancer Center, Houston, TX, USA

^5^Department of Rheumatology and Rehabilitation, Faculty of Medicine, Assiut University Hospitals, Assiut, Egypt

^6^Department of Epidemiology, Division of Cancer Prevention and Population Sciences, The University of Texas MD Anderson Cancer Center, Houston, TX, USA

^7^Department of Gastroenterology, Hepatology, and Nutrition, Division of Internal Medicine, The University of Texas MD Anderson Cancer Center, Houston, TX, USA
