# Supplementary material for: Infliximab associated with faster symptom resolution compared with corticosteroids alone for the management of immune-related enterocolitis
Source: J Immunother Cancer. 2018 Oct 11;6:103. doi: 10.1186/s40425-018-0412-0 (PMC6180568; doi:10.1186/s40425-018-0412-0)
Supplement: Supplementary file 1 — Figure S1. Kaplan-Meier analysis of time to treatment failure in patients with stage IV melanoma. Median TTF was 9.0 months (95% CI 5.6 months–not reached) in the IFX group and 12.5 months (95% CI 5.8 months–not reached) in the CS group. Median follow-up 26 months. (DOCX 48 kb) [file 40425_2018_412_MOESM1_ESM.docx]

**
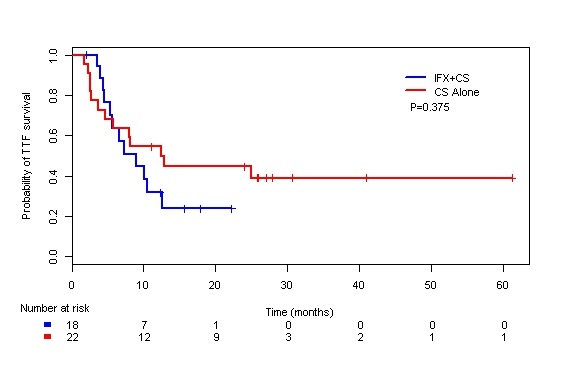
Additional file 1 Figures/Tables**

***Additional file 1: Figure S1:* Kaplan-Meier analysis of time to treatment failure in patients with stage IV melanoma.**

Median TTF was 9.0 months (95% CI 5.6 months–not reached) in the IFX group and 12.5 months (95% CI 5.8 months–not reached) in the CS group. Median follow-up 26 months

**Additional file 1: Table S1: Adverse Events Related To Treatment**

| Adverse Event | Overall (N=75) | Infliximab + CS (N=36) | CS (N=39) | P Value |
| --- | --- | --- | --- | --- |
| CS-Related, No. (%) |  |  |  |  |
| All | 40 (53.3) | 22 (61.1) | 18 (46.2) | 0.249 |
| Hyperglycemia | 23 (30.7) | 14 (38.9) | 9 (23.1) | 0.210 |
| Muscle Weakness | 10 (13.3) | 6 (16.7) | 4 (10.3) | 0.506 |
| Edema | 4 (5.3) | 1 (2.8) | 3 (7.7) | 0.616 |
| Psychosis | 2 (2.7) | 2 (5.6) | 0 | 0.227 |
| Fatigue | 3 (4.0) | 1 (2.8) | 2 (5.1) | 1.000 |
| Other  Insomnia  Nausea/Vomiting  GI Bleed  Hypertension  Increased Appetite  Infection | 10 (13.3)  4 (5.3)  1 (1.3)  1 (1.3)  1 (1.3)  1 (1.3)  2 (2.7) | 5 (13.9)  1 (2.8)  1 (2.8)  1 (2.8)  1 (2.8)  0 (0)  0 (0) | 5 (12.8)  3 (7.7)  0 (0)  0 (0)  0 (0)  1 (2.6)  2 (5.1) | 1.000 |
| Infliximab-Related, No. (%) |  |  |  |  |
| All | 3 (4.0) | 3 (8.3) |  |  |
| Herpes | 1 (1.3) | 1 (2.8) |  |  |
| Cytomegalovirus Viremia | 1 (1.3) | 1 (2.8) |  |  |
| Diaphoresis | 1 (1.3) | 1 (2.8) |  |  |

CS=corticosteroids
